# MitoNEET Provides Cardioprotection via Reducing Oxidative Damage and Conserving Mitochondrial Function

**DOI:** 10.3390/ijms25010480

**Published:** 2023-12-29

**Authors:** Eddie Tam, Gary Sweeney

**Affiliations:** Department of Biology, York University, Toronto, ON M3J 1P3, Canada

**Keywords:** mitoNEET, mitochondria, iron metabolism, oxidative stress, cardiometabolic disease

## Abstract

Cardiometabolic diseases exert a significant health impact, leading to a considerable economic burden globally. The metabolic syndrome, characterized by a well-defined cluster of clinical parameters, is closely linked to an elevated risk of cardiovascular disease. Current treatment strategies often focus on addressing individual aspects of metabolic syndrome. We propose that exploring novel therapeutic approaches that simultaneously target multiple facets may prove more effective in alleviating the burden of cardiometabolic disease. There is a growing body of evidence suggesting that mitochondria can serve as a pivotal target for the development of therapeutics aimed at resolving both metabolic and vascular dysfunction. MitoNEET was identified as a binding target for the thiazolidinedione (TZD) class of antidiabetic drugs and is now recognized for its role in regulating various crucial cellular processes. Indeed, mitoNEET has demonstrated promising potential as a therapeutic target in various chronic diseases, encompassing cardiovascular and metabolic diseases. In this review, we present a thorough overview of the molecular mechanisms of mitoNEET, with an emphasis on their implications for cardiometabolic diseases in more recent years. Furthermore, we explore the potential impact of these findings on the development of novel therapeutic strategies and discuss potential directions for future research.

## 1. Introduction

### 1.1. Cardiovascular Diseases

Cardiovascular events, including ischemic heart disease, stroke, atherosclerosis, and heart failure, stand as prominent contributors to global mortality [1]. The pathogenesis of cardiovascular diseases is remarkably diverse, involving a myriad of mechanisms, two prominent contributors being oxidative stress and cell death [2]. During oxidative stress, there is an imbalance in the redox state of cells due to the increased production of reactive oxygen species (ROS). ROS refers to oxygen radicals such as hydroxyl radicals, superoxide, and peroxyl radicals, in addition to non-radicals such as hydrogen peroxide [3]. Numerous endogenous mechanisms exist to counteract oxidative stress; however, when the production of reactive oxygen species (ROS) surpasses the antioxidant capacity, it can significantly contribute to the development of cardiovascular diseases [4]. Cell death is one of the downstream events triggered by oxidative stress which can contribute to cardiovascular diseases [2,5]. Due to the limited regenerative capacity of the adult heart, even a minor degree of cell death can have a profound and detrimental impact on cardiac function [6].

### 1.2. Metabolic Diseases

The prevalence of metabolic diseases is on the rise, paralleled by an increase in associated mortality [7]. As an example, the mortality linked to obesity was 5 million in 2019 and is anticipated to more than double by 2050 [7,8]. Metabolic diseases are intricately connected to cardiovascular diseases [9]. The linkage between metabolic diseases and cardiovascular issues is underscored by the widespread recognition of metabolic syndrome (MetS) whose definition has been refined and a global consensus definition has been established by the IDF [10]. According to that definition, metabolic syndrome is characterized by the presence of obesity along with any two of the following criteria: elevated serum triglycerides, decreased serum HDL cholesterol, glucose intolerance, and hypertension. Each of these factors is acknowledged as a risk factor for cardiovascular diseases.

### 1.3. MitoNEET as a Potential Therapeutic Target

While there have been advancements in therapeutics for cardiovascular and metabolic diseases, their efficacy has not kept pace with the growing demand for innovation, especially as new insights into disease mechanisms continue to emerge [11]. Moreover, the development of novel therapeutics targeting mechanisms at the intersection of cardiovascular and metabolic outcomes is likely to have a more impactful and successful effect on patients grappling with these chronic diseases.

The interest in developing mitochondrial-targeted therapeutics is on the rise, driven by the association of dysfunctional mitochondria with various common pathologies [12]. This is further exemplified by the growing interest in the therapeutic potential of mitoNEET—a transmembrane protein located in the outer mitochondrial membrane named after a conserved amino acid sequence, part of which includes Asn-Glu-Glu-Thr (NEET) [13,14,15,16,17,18,19]. It was originally identified as an unknown, yet high-affinity binding target of the antidiabetic TZD drug, pioglitazone [20]. Since its initial discovery in 2004, the protein has been explored as a potential therapeutic target in various conditions such as neurological disorders, cancer, cardiovascular diseases, and metabolic diseases [13,15,18,19,21,22,23,24].

**Figure 1:**
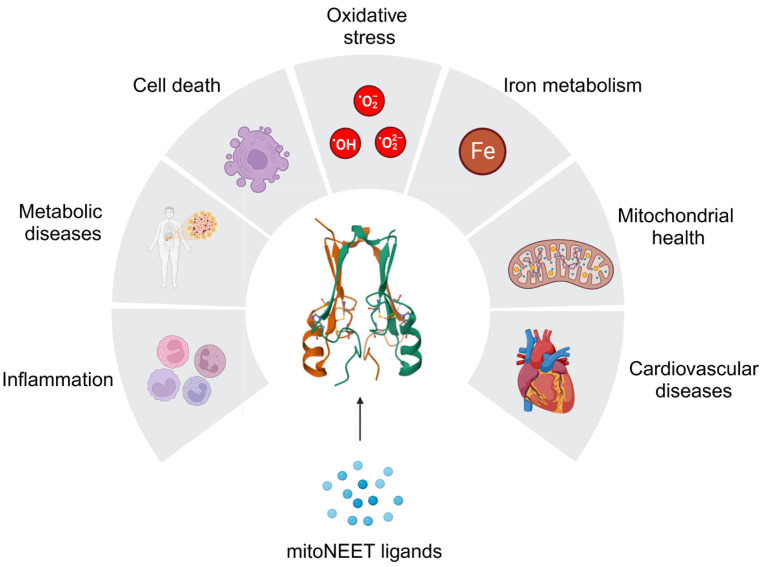
MitoNEET regulates a wide array of biological processes.

In the following review, we aim to highlight recent advances in elucidating the function of the mitoNEET protein, with an emphasis on its relevance to cardiovascular and metabolic diseases (Figure 1). Furthermore, we aim to identify gaps in the current knowledge to guide future investigations which will accelerate the development of improved therapeutic strategies. 

MitoNEET plays a role in regulating inflammation, metabolic diseases, cell death, oxidative stress, iron metabolism, mitochondrial health, and cardiovascular diseases. The dysregulation of any of these processes has been implicated in the development of metabolic and cardiovascular diseases. Targeting the mitoNEET protein can be accomplished using mitoNEET ligands such as pioglitazone, TT01001, NL-1, MGZ, and TZDs. The illustration was made using biorender.com.

## 2. Mitochondrial Homeostasis and mitoNEET

### 2.1. MitoNEET, Mitochondria, and Metabolic Diseases

**Figure 2:**
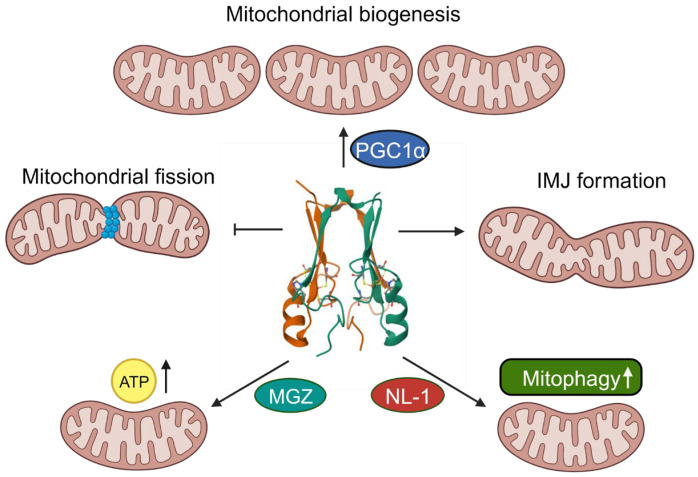
MitoNEET in mitochondrial homeostasis.

Thiazolidinediones (TZDs) are a well-established class of anti-diabetic drugs that provide an insulin-sensitizing effect [25]. Although the biological effects of TZDs were once thought to be mediated via peroxisome proliferator-activated receptor gamma (PPARγ) signaling, it is now known that they can exert a biological effect independent of PPARγ [26]. The insulin-sensitizing effects of TZDs are now attributed, in part, to the involvement of mitochondrial proteins, including mitoNEET, and peroxisome proliferator-activated receptor-gamma coactivator (PGC)1α [27]. As such, these mitochondrial proteins may prove to have the potential for developing future therapeutics relating to metabolic diseases. Indeed, various studies employing genetic manipulation to alter mitoNEET levels have demonstrated its involvement in crucial mitochondrial processes, including respiration, biogenesis, and quality control through mitophagy. These processes are known to be altered in pathological states [28,29,30]. Collectively, this justifies exploring mitoNEET as a potential therapeutic target in mitochondrial dysfunction and related pathologies. The various mitochondrial processes affected by mitoNEET are summarized in Figure 2 and are described further in subsequent sections.

MitoNEET promotes mitochondrial homeostasis by regulating mitochondrial morphology through the formation of intermitochondrial junctions (IMJ) and inhibition of mitochondrial fission. MitoNEET also upregulates PGC1α to mediate an increase in biogenesis. Mitoglitazone (MGZ) and novel mitoNEET ligand 1 (NL-1) increase ATP production and mitophagy, respectively, via mitoNEET. The illustration was made using Biorender.com.

### 2.2. MitoNEET Regulates Mitochondrial Morphology

Mitochondrial dynamics, encompassing the processes of fusion and fission that determine mitochondrial morphology, play a crucial role in the development of insulin resistance [31]. Mitochondrial fusion and fission occur simultaneously in an equilibrium under physiological conditions which can become disrupted in pathological states [31]. For instance, a shift towards increased fission, leading to more fragmented mitochondria, has been implicated in the development of insulin resistance. Inhibiting this fission process holds therapeutic potential for addressing insulin resistance [32]. Interestingly, mitoNEET has been shown to play a role in regulating mitochondrial dynamics. Overexpression of mitoNEET has been observed to mitigate mitochondrial fragmentation under conditions of iron overload by suppressing the upregulation of the fission-related protein, Fis1 [18]. Moreover, the expression of a mutant form of mitoNEET with enhanced resistance to oxidative stress resulted in reduced hydrogen peroxide-induced fragmentation [33]. Additionally, mitoNEET plays a role in regulating mitochondrial networks independently of the fusion/fission machinery by promoting the formation of intermitochondrial junctions (IMJs). Notably, mitoNEET can increase IMJs even in the absence of fusion-related proteins such as mitofusin ½ [33]. Knockout of mitoNEET reduced IMJ and mitochondrial volume [33]. Indeed, the evidence suggests that mitoNEET plays a crucial role in regulating mitochondrial morphology through the formation of IMJs and influencing mitochondrial dynamics. These processes that alter mitochondrial morphology have direct implications for insulin resistance.

### 2.3. Mitochondrial Health and MitoNEET

MitoNEET is integral to maintaining mitochondrial function. In instances such as mitoNEET null mice, there is a notable reduction in ATP production, highlighting the importance of mitoNEET in sustaining mitochondrial energy output [22]. Similarly, cardiac-specific loss of mitoNEET reduced mitochondrial respiration and ATP levels [21]. Wiley et al. found that loss of mitoNEET resulted in a decrease of maximal (state 3u) respiration rates, indicating that mitoNEET may serve an important role during a physiological energy demand [30]. This suggests that mitoNEET plays a crucial role in maintaining mitochondrial respiration and ensuring an adequate supply of ATP. Yonutas et al. demonstrated that the use of pioglitazone improved mitochondrial function, proving beneficial in their models of neurodegenerative diseases [19]. Separate studies also found that other mitoNEET ligands such as NL-1 and mitoglitazone (MGZ—a derivative of pioglitazone with higher binding affinity for mitoNEET and reduced side effects) improved mitochondrial health through mitophagy and biogenesis with increase in ATP production [16,29,34]. Mitophagy is an important quality control process for eliminating damaged mitochondria and its dysregulation is implicated in the development of insulin resistance [35]. Following mitophagy, the biogenesis of new mitochondria is important to replenish healthy mitochondrial content necessary for cell survival [36]. The capacity of various mitoNEET ligands to enhance mitophagy and mitochondrial biogenesis suggests that mitoNEET could be a pivotal regulator of quality control processes. Therefore, this underscores mitoNEET’s crucial role in maintaining mitochondrial health and positions it as a promising pharmacological target for pathologies associated with mitochondrial dysfunction.

## 3. Iron Metabolism and MitoNEET

### 3.1. The Role of Dysregulated Iron Metabolism in Cardiovascular and Metabolic Diseases

The regulation of iron is crucial because an excess has been associated with disease states, given its ability to undergo reactions producing toxic byproducts, mitochondrial disturbances, and subsequent pathologies [37]. Since iron is an essential micronutrient, a nonspecific reduction in whole-body iron levels may have adverse effects. In fact, the use of iron chelators has been associated with a diverse range of side effects, including bone abnormalities and neurological disorders [38]. Moreover, specific iron chelators, like deferoxamine, exhibit low cell permeability, making them effective at targeting circulating iron but less so at reaching intracellular iron stores [39].

There is increasing evidence indicating mitochondria-specific iron, rather than total iron, is the key to disease progression [40,41,42]. In fact, the selective reduction of mitochondrial iron has been shown to be therapeutic in certain cardiovascular and metabolic diseases [18,40,41], however the use of pharmacological agents targeting mitochondrial iron beyond an in vitro setting has largely been unexplored [43]. Interestingly, the use of the GLP-1 receptor agonist (GLP-1-RA) exenatide (used to treat diabetes) has been found to prevent mitochondrial iron accumulation as effectively as the iron chelator deferiprone [44]. Therefore, targeting mitochondria-specific iron may prove to be an effective therapeutic strategy while at the same time, overcoming challenges associated with classical iron chelation therapy.

### 3.2. MitoNEET and Iron Metabolism

**Figure 3:**
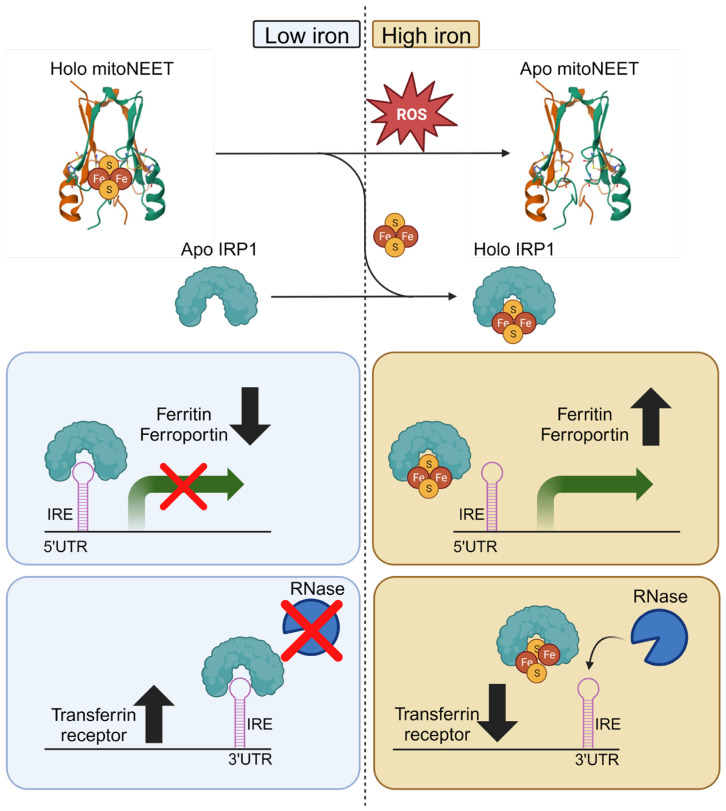
Mechanism of iron regulation by mitoNEET ISC.

Iron regulatory protein 1 (IRP1) is an important regulator of iron homeostasis through the regulation of genes involved in iron uptake, storage, and export [45]. Upon exposure to oxidative stress, IRP1 can be damaged and converted to the apo (iron-free)-form and mitoNEET has the capacity to repair IRP1 converting it from the apo- to the holo (iron-bound)-form [46]. The holo-form of mitoNEET, containing an iron-sulfur cluster (ISC), is resistant to oxidative stress and can transfer its ISC to damaged IRP1 [46]. MitoNEET plays a crucial role in cytosolic aconitase activity by mediating the transfer of its ISC to aconitase [46]. Additionally, this repair process is particularly significant following oxidative damage, given mitoNEET’s high resistance to hydrogen peroxide in comparison to other ISC-containing proteins [47]. It is conceivable that mitoNEET may contribute to the repair of other ISC-containing proteins, although this hypothesis remains to be tested. Therefore, the interaction with the ISC repair pathway could potentially contribute to mitoNEET’s ability to modulate iron homeostasis (Figure 3).

During conditions of low iron, such as in physiological conditions, mitoNEET exists in its holo-form bound to ISC. Iron regulatory protein 1 (IRP1) exists in its apo form with iron response element (IRE) binding ability. IRP1 binding to IRE within the 5′UTR results in translational repression (ferritin and ferroportin), whereas binding to IRE within the 3′UTR confers mRNA stability from RNase degradation (transferrin receptor). In contrast, under conditions of high iron resulting in oxidative stress such as in pathological conditions, mitoNEET can transfer its ISC to IRP1 which lacks IRE binding ability. As a result, the translation of ferritin and ferroportin is upregulated due to a loss of IRP1 repression. The transferrin receptor is downregulated due to loss of mRNA stability. This illustration was made using Biorender.com

### 3.3. MitoNEET Regulates Mitochondrial Iron

MitoNEET can alter mitochondrial iron content in various settings. For example, adipose tissue-specific overexpression of mitoNEET by approximately five-fold in mice (mitoNEET-Tg mice) led to a 50% reduction in mitochondrial iron content compared to wild-type (WT) mice [15]. Additionally, mitoNEET was observed to be upregulated in mice subjected to a high iron diet and in a genetic model of iron overload known as hemochromatosis [15]. This suggests that mitoNEET expression may increase as a compensatory effect to avert excess mitochondrial iron. Indeed, upon feeding mice a high iron diet, there were increases in mitochondrial iron content, and this elevation was mitigated in mitoNEET-Tg mice, where mitoNEET was overexpressed in adipose tissue. The reduced mitochondrial iron in mitoNEET-Tg mice also correlated with reduced ROS and heightened insulin sensitivity [15]. Similar findings were reported in an in vitro setting using cardiac cells, where overexpression of mitoNEET preserved mitochondrial iron homeostasis. This preservation correlated with protective effects against mitochondrial reactive oxygen species (ROS) formation, apoptosis [17], and insulin resistance [18]. Conversely, the cardiac-specific knockout of mitoNEET in mice led to elevated mitochondrial iron content [21]. Mitochondrial ferritin, an iron storage protein used as an indicator of mitochondrial iron overload, was also elevated upon knockout of cardiac mitoNEET [21]. Despite this, known regulators of mitochondrial and cellular iron homeostasis were unchanged by mitoNEET knockout [21]. This indicates that mitoNEET plays a role in mitochondrial iron homeostasis independent of canonical iron homeostatic pathways. Hence, mitoNEET demonstrates the capacity to inhibit excess mitochondrial iron accumulation in various tissue types, both in vivo and in vitro, and exhibits a protective effect in pathological states. However, in these studies, whether mitoNEET regulates iron homeostasis via interactions with the ISC repair pathway remains to be determined.

## 4. MitoNEET and Redox Homeostasis

### 4.1. Limitations in Using Antioxidants as Therapeutics

The modulation of oxidative stress as a treatment for cardiovascular diseases and metabolic disorders has demonstrated some potential [48,49]. Nevertheless, existing antioxidant therapies for cardiovascular and metabolic diseases have some drawbacks. The lack of specificity in certain anti-oxidative therapeutic agents can lead to off-target effects, causing unexpected side effects in a clinical setting. For example, the use of sapropterin has been associated with NOS uncoupling, a phenomenon that paradoxically exacerbates oxidative stress and is linked to the development of cardiovascular diseases [50,51,52]. The broad-spectrum antioxidant N-acetyl cysteine (NAC), functioning as a reactive oxygen species (ROS) scavenger and serving as a precursor for the generation of the glutathione (GSH) antioxidant pool, has shown some cardioprotective and improved metabolic effects [53,54]. Although there has been success with N-acetyl cysteine (NAC) in animal models, its application in humans has faced challenges, primarily due to difficulties in achieving optimal dosages and the occurrence of anaphylactoid reactions [54,55,56]. Consequently, the use of antioxidants for cardiometabolic diseases in humans remains a topic of controversy. Therapeutic strategies focusing on enhancing endogenous antioxidant capacity are anticipated to yield more promising outcomes, but current approaches necessitate further refinement and improvement.

### 4.2. MitoNEET Iron-Sulfur Cluster Status Acts as a Redox Sensor

Indeed, mitoNEET has demonstrated its involvement in regulating the redox state. A deeper understanding of mitoNEET could potentially contribute to the enhancement of current antioxidative therapies. The ISC associated with mitoNEET is capable of being released and donated to acceptor proteins, serving as an indicator of its redox state. Importantly, the ISC can undergo both reduction and oxidation processes [57]. In situations of oxidative stress, the ISC bound to mitoNEET is readily released, potentially serving as a signaling event that indicates the presence of oxidative stress [47,57]. The use of TZDs such as pioglitazone represses the oxidative stress signal by stabilizing and preventing the release of the ISC from mitoNEET [58]. The mitoNEET protein can also undergo reduction facilitated by antioxidant species such as glutathione, glutathione reductase, N-acetyl cysteine, and l-cysteine. This reduction process stabilizes the ISC associated with mitoNEET [59]. MitoNEET is more resistant to ROS such as hydrogen peroxide compared to other ISC transfer proteins making it suitable to function as a redox sensor [47]. Collectively, these observations suggest that during conditions of oxidative stress, there is a loss of the ISC from mitoNEET. In contrast, when oxidative stress is attenuated, the interaction between ISC and mitoNEET is stabilized (Figure 3). Hence, the lability of the ISC on mitoNEET can serve as an indicator of oxidative stress.

### 4.3. MitoNEET Mitigates Oxidative Stress

**Figure 4:**
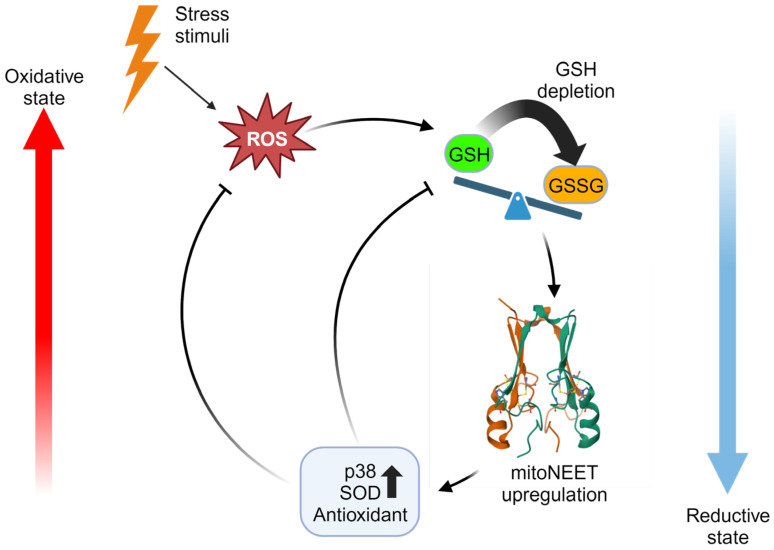
Regulation of oxidative stress by mitoNEET.

Manipulation of mitoNEET expression has revealed its potential antioxidative properties, which can have profound effects in regulating oxidative stress. MitoNEET overexpression in both cells and animal models has been shown to reduce levels of reactive oxygen species (ROS) under pathological conditions [15,17,18]. Furthermore, mitoNEET played a role in promoting the survival of cardiac stem cells in an oxidative stress environment [60]. MitoNEET is also now known to interact with the well-known GSH antioxidant system [23]. Inhibition of glutathione (GSH) reductase in cardiomyocytes with 2-acetylamino-3-[4-(2-acetylamino-2-carboxyethylsulfanylthio-carbonylamino) phenylthiocarbamoylsulfanyl] propionic acid (2-AAPA) decreased GSH/GSSG ratio, suggestive of oxidative stress [23]. Unexpectedly, 2-AAPA reduced oxidative stress-induced cell death and increased mitoNEET levels, due to the inhibition of GSH recycling. Supplementation with exogenous GSH reversed this effect [23]. This shows that mitoNEET compensates for depletion in the GSH antioxidant pool and can serve to protect the heart against oxidative injury. In a related context, the overexpression of mitoNEET has been demonstrated to confer resistance to oxidative stress in the nematode Caenorhabditis elegans (C. elegans) [61]. Activation of the p38 signaling pathway serves as a defense mechanism against environmental stress, including oxidative stress. When challenged with paraquat-induced oxidative stress, the overexpression of mitoNEET enhanced p38 phosphorylation, indicating its potential role in modulating the cellular response to oxidative stress [61]. MitoNEET antioxidative properties are further highlighted by its ability to induce upregulation of superoxide dismutase 2 (SOD2) [29,61]. The reduction of mitoNEET expression by isoliquiritigenin (ISL) leads to an increase in reactive oxygen species (ROS), while the overexpression of mitoNEET counteracts ISL-induced ROS, suggesting a regulatory role for mitoNEET in oxidative stress modulation [13]. Targeting mitoNEET with NL-1 has been found to be protective against ischemia-reperfusion injury in brain endothelial cells induced by peroxide generation. This underscores the potential therapeutic role of mitoNEET modulation in mitigating oxidative stress-related damage [24]. Similarly, the mitoNEET ligand, TT01001, reduced oxidative stress in a model of brain injury [62,63]. The use of pioglitazone reduced ferroptosis induction by erastin due to the mitigation of oxidative damage by stabilizing the ISC on mitoNEET [64]. Indeed, mitoNEET plays a critical role in regulating oxidative stress. Beyond its function as a redox sensor, it can directly mediate antioxidative properties (Figure 4). Given the close association between oxidative stress and cardiovascular and metabolic diseases, gaining further insights into mitoNEET may hold the potential for advancements in current antioxidant therapies.

The endogenous GSH/GSSG antioxidant system mitigates oxidative stress by scavenging ROS. Upon depletion of the reduced GSH, levels of mitoNEET are upregulated as a compensatory mechanism. MitoNEET inhibits ROS, in part through upregulation of p38 and superoxide dismutase (SOD). The illustration was made using Biorender.com.

## 5. Cardioprotective Potential of MitoNEET via Mitigating Cell Death

### 5.1. Implications of Cell Death on Cardiovascular Diseases

The death of cardiomyocytes has been implicated in cardiovascular diseases, including heart failure [65]. The use of caspase inhibitors has demonstrated some cardioprotective potential via their antiapoptotic mechanisms [66]. However, the progress in the clinical application of certain approaches, such as the risk of tumor induction and poor specificity, has been greatly limited [66]. Hence, a deeper understanding of alternative targets involved in the regulation of apoptosis may provide insights to overcome limitations associated with current therapeutic approaches.

### 5.2. MitoNEET Regulates Apoptosis in Cardiomyocytes and Cardiomyoblasts

In the cardiac setting, mitoNEET holds potential therapeutic implications for cardiovascular diseases by regulating cell death. For example, in a model of hypoxia/reoxygenation (H/R) in HL-1 cardiac cells, mitoNEET was observed to provide protection against oxidative stress-induced apoptosis [23]. Specifically, the knockdown of mitoNEET increased H/R-induced apoptosis. Conversely, pharmacological manipulation of the antioxidant system to deplete GSH levels resulted in a 4-fold increase in mitoNEET protein which reduced H/R-induced apoptosis [23]. In a pair of studies utilizing an in vitro oxygen-glucose deprivation model to mimic ischemia/reperfusion, the application of mitoNEET ligand NL-1 demonstrated a reduction in peroxide generation and subsequent apoptosis [24,34]. Moreover, mitoNEET was also shown to be protective against apoptosis through the regulation of mitochondrial iron in H9c2 cardiac cells [17]. Excess mitochondrial iron overload (IO) was found to induce upregulation of mitochondrial ROS, leading to cytochrome c release, and ultimately apoptosis. The overexpression of mitoNEET averted the accumulation of mitochondrial iron, which prevented the induction of IO-induced apoptosis [17]. Therefore, mitoNEET can play a protective role against cell death under conditions of stress and this has relevance to therapeutic potential for cardiovascular diseases.

### 5.3. MitoNEET Reduces Cell Death via Supression of Ferroptosis

Ferroptosis is a form of cell death triggered by lipid peroxidation and can occur independently of apoptosis [67]. There is a growing body of evidence indicating that ferroptosis plays a role in mediating cardiovascular diseases, and targeting ferroptosis remains a promising therapeutic strategy [42]. Given its involvement in ferroptosis, mitoNEET may possess therapeutic potential for cardiovascular diseases. The role of mitoNEET in ferroptosis has been investigated using erastin, a well-established inducer of ferroptosis that blocks cystine uptake [64]. Interestingly, erastin increased mitoNEET gene expression in a dose-dependent manner [64]. RNAi-mediated knockdown of mitoNEET exacerbated erastin-induced mitochondrial iron accumulation, lipid peroxidation, and ferroptosis [64]. Pioglitazone reduced erastin-induced accumulation of mitochondrial iron, mitochondrial lipid peroxidation, and cell death [64]. This suggests mitoNEET can mitigate ferroptosis, possibly by inhibiting lipid peroxidation and/or iron accumulation. Similarly, targeting mitoNEET using MGZ demonstrated a protective effect against erastin-induced ferroptosis and improved cell viability in HEK293 cells [16]. Additionally, MGZ attenuated non-heme iron accumulation, lipid peroxidation, and depletion of glutathione levels in mice subjected to kidney ischemia-reperfusion injury [16]. The evidence implicating mitoNEET as a regulator of ferroptosis adds further to its attraction as a therapeutic target for cardiovascular diseases.

### 5.4. Physiological Applications of MitoNEET in Cardiovascular Disease

Stem cell therapy has garnered increasing attention as a therapeutic strategy for cardiovascular diseases [68]. One of the limitations of stem cell therapy is the challenge of maintaining the viability of stem cells in an oxidative environment, which is a hallmark of ischemic heart disease [60]. However, because mitoNEET plays a role in regulating oxidative stress, it may aid in improving the efficacy of stem cell therapy. For example, the pharmacological manipulation of mitoNEET with NL-1 has been shown to enhance the survival of cardiac stem cells subjected to exogenous hydrogen peroxide, simulating an in vitro oxidative stress environment [60]. The protective effects of NL-1 were not observed in hydrogen peroxide-treated cardiac stem cells isolated from mitoNEET knockout mice. This validates the specificity of NL-1 for the mitoNEET protein [60]. Moreover, when cardiac stem cells were intramyocardially injected into Zucker obese fatty rats, their survival increased when the rats were treated with NL-1 [60]. Therefore, mitoNEET could be crucial in developing enhancements for the effectiveness of stem cell therapy in the cardiac setting. Furthermore, mitoNEET has been shown to play an important role in the pathogenesis of age-related heart failure. A decrease in mitoNEET has also been associated with age-related heart failure, as mitoNEET expression is downregulated in the heart during the aging of mice [21]. Moreover, cardiac-specific deletion of mitoNEET in mice led to left ventricular dysfunction and heart failure at younger ages compared to control mice [21]. This poor prognosis was associated with abnormal mitochondrial morphology, reduced respiration, and elevated oxidative stress [21]. This finding is intriguing, as heart failure predominantly affects the elderly, with an average age of diagnosis around 76. However, geriatric patients are often under-represented in major heart failure clinical trials, where instead, younger adults are more commonly studied [69,70]. Whether this underrepresentation interferes with our understanding of the contribution of the loss of mitoNEET expression in the heart during chronological aging will be of interest to investigate. Thus, the loss of mitoNEET in the aging heart has been implicated in heart failure and death. It is possible that TZDs or NL-1 could prove to have therapeutic efficacy in the aging population.

## 6. Metabolic Effects of MitoNEET in Cardioprotection

### 6.1. MitoNEET Preserves Metabolic Health in Obesity

Metabolic dysfunction and cardiovascular diseases are intricately linked, a connection reinforced by the heightened risk for adverse cardiovascular events in the presence of metabolic syndrome, as described earlier. There is evidence linking reduced mitoNEET levels with metabolic dysfunction. For example, levels of mitoNEET expression are reduced when challenged by high-fat diet (HFD)-induced obesity in mice [15]. To investigate the relationship between mitoNEET and metabolism in an in vivo setting, Kusminski et al. created a transgenic mouse model of adipose-specific mitoNEET overexpression (mitoNEET-Tg) on an ob/ob genetically obese background [15]. Intriguingly, mitoNEET-Tg obese mice exhibited reduced fatty acid oxidation and increased triglyceride (TAG) storage in adipose tissue, resulting in increased obesity. However, despite the increased adiposity, these mice showed improved insulin sensitivity and glucose tolerance compared to non-transgenic obese ob/ob mice [15]. This improved insulin sensitivity was associated with increased adiponectin mRNA and protein [15]. Furthermore, mitoNEET-Tg obese mice demonstrated enhanced TAG clearance from the serum, coupled with reduced lipid accumulation in the liver. This pattern is consistent with increased storage of lipids in adipose tissue but with benign consequences [15]. As indicated above, mitoNEET was also shown to reduce mitochondrial iron and oxidative stress and increase adiponectin production [15]. Conversely, when there was a whole-body reduction of mitoNEET in adipose and other tissues in C57BL/6 mice, it led to higher mitochondrial iron levels, increased mitochondrial oxidative capacity, elevated oxidative damage to proteins (ROS), and impaired glucose tolerance. This occurred despite a reduction in the storage of lipids in both adipose tissue and the liver when the mice were fed a high-fat diet [15]. Therefore, manipulation of mitoNEET expression in adipocytes had beneficial impacts on whole-body metabolism.

Additional studies using adipose-specific mitoNEET-Tg mice revealed that mitoNEET promoted an anti-inflammatory environment in adipose tissue, enriched with M2 macrophages, as opposed to the pro-inflammatory M1 macrophages typically observed in an obese phenotype [28]. MitoNEET was found to promote a healthy expansion of white adipose tissue (WAT) with age, contributing to the prevention of lipotoxicity-induced insulin resistance. The mitoNEET-Tg model represents a metabolically healthy, yet obese (MHO) phenotype, akin to upwards of 40% of the obese population observed in human studies [71]. Despite its identification in the clinical setting, the MHO paradox is not fully understood. It would be of interest to determine if elevated mitoNEET could be an underlying factor of this MHO subset within the obese population. 

### 6.2. MitoNEET as a Therapeutic Target in Metabolic Dysfunction

Targeting mitoNEET has shown potential as a therapeutic approach in diabetes. In an in vivo model of type 2 diabetes (T2D) using db/db mice, the mitoNEET ligand TT01001 improved hyperglycemia and glucose intolerance [63]. The therapeutic effects of TT01001 on glucose metabolism were comparable to those of pioglitazone, but without the associated side effect of weight gain [63]. Furthermore, in H9c2 cardiac cells, the overexpression of mitoNEET was protective against a model of IO-induced insulin resistance through the regulation of mitochondrial iron and ROS [18]. MitoNEET may also play a role in glucose homeostasis through its interaction with glutamate dehydrogenase 1 (GDH1) as a binding partner [72]. GDH1 has been known to play a role in insulin secretion, especially in a high glucose setting [73], suggesting mitoNEET could enhance GDH1 activity to aid insulin secretion and maintain glucose homeostasis. Collectively, these findings further support mitoNEET as a potentially useful therapeutic target in the treatment of metabolic diseases such as diabetes.

### 6.3. MitoNEET in Regulating Crosstalk between Adipose and Cardiac Tissue

The connection between metabolic diseases and cardiovascular diseases, as described earlier, is further demonstrated by the ability of adipose and cardiac tissues to crosstalk [74]. Under normal conditions, perivascular adipose tissue (PVAT) secretes anti-atherogenic signals [75]. However, in certain disease states, PVAT can promote atherosclerosis through the secretion of proinflammatory molecules [75]. Elevated angiotensin II is the main atherogenic component of the renin-angiotensin-aldosterone system [76]. Angiotensin II administration in mice elevated inflammation and reduced browning in PVAT [76]. Conversely, PVAT from mitoNEET-Tg mice exhibits a more brown-like phenotype with some resistance to angiotensin II-induced inflammation [76]. Moreover, the brown adipose-specific overexpression of mitoNEET resulted in perivascular adipose tissue (PVAT) with increased thermogenesis, an anti-inflammatory response, and prevention of the development of atherosclerosis upon high-fat diet (HFD) feeding [77]. Therefore, mitoNEET has been shown to play a role in the metabolic reprogramming of perivascular adipose tissue (PVAT) to counterbalance angiotensin II-induced proinflammatory status in PVAT. Additionally, mitoNEET has also been demonstrated to play a role in arterial stiffening, a known consequence of aging and an early indicator of cardiovascular disease [78]. HFD feeding of aged mice was found to induce hypertrophy in PVAT, arterial stiffness, and reduce mitoNEET expression [78]. In contrast, brown adipose-specific mitoNEET overexpression elevated mitoNEET expression in the PVAT, and this negatively correlated with inflammation and arterial stiffness [78]. Thus, targeting mitoNEET in PVAT may tackle issues at the interface of both cardiovascular and metabolic diseases and give rise to the development of new preventative therapeutics.

## 7. Conclusions

The current understanding of the mitoNEET protein justifies exploring it as a novel target for the treatment of cardiovascular and metabolic diseases. At the molecular level, mitoNEET has been implicated in regulating oxidative stress, iron metabolism, and mitochondrial homeostasis. MitoNEET plays a role in regulating mitochondrial morphology via the formation of IMJs, inhibits mitochondrial fragmentation, enhances ATP production, and improves quality control processes. Oxidative stress can be detected by redox sensors such as mitoNEET, which subsequently enhances endogenous antioxidants, namely glutathione (GSH). Furthermore, mitoNEET also serves an important function in the development of cardiovascular and metabolic diseases. MitoNEET promotes cardiac cell survival under conditions of stress and has been protective against age-related heart failure through its anti-apoptotic and anti-ferroptotic effects. Genetic or pharmacological approaches targeting mitoNEET have been demonstrated to be protective against IO-induced and obesity-induced insulin resistance. Similarly, the mitoNEET ligand TT01001 was beneficial for hyperglycemia and glucose intolerance. MitoNEET may also underlie the rise of the metabolically healthy obese (MHO) phenotype by altering whole-body metabolism to avoid lipotoxicity, ultimately preserving insulin sensitivity. Finally, mitoNEET may play a role in mediating the intersection between cardiovascular diseases and metabolic diseases by regulating the metabolic profile of perivascular adipose tissue (PVAT). In conclusion, mitoNEET plays a crucial function in preventing the development of cardiovascular and metabolic diseases, and a deeper understanding will allow for the advancement of improved therapeutic strategies.

## Data Availability

Not applicable.
